# Impact of the Enzyme Charge on the Production and Morphological Features of Cellulose Nanofibrils

**DOI:** 10.3390/polym13193238

**Published:** 2021-09-24

**Authors:** Sergio Henríquez-Gallegos, Gregory Albornoz-Palma, Andrea Andrade, Carolina Soto, Miguel Pereira

**Affiliations:** 1 Departamento de Ingeniería Química, Facultad de Ingeniería, Universidad de Concepción, Concepción 4030000, Chile; sergiohenriquez@udec.cl (S.H.-G.); gralbornoz@udec.cl (G.A.-P.); carosotoh@udec.cl (C.S.); 2 Facultad de Ciencias Forestales, Universidad de Concepción, Concepción 4030000, Chile; anandrade@udec.cl; 3 Unidad de Desarrollo Tecnológico (UDT), Universidad de Concepción, Coronel 4190000, Chile

**Keywords:** cellulose nanofibril, enzymatic hydrolysis, degree of polymerization, fibrillation process, morphology

## Abstract

The available research does not allow specific relationships to be established between the applied enzymatic-mechanical treatment, the degree of polymerization, and the characteristics of the cellulose nanofibrils (CNFs) produced. This work aims to establish specific relationships between the intensity of enzymatic treatment, the degree of polymerization of the cellulose, the morphology of CNFs, and the tensile strength of the CNF films. It is determined that the decrease in the degree of polymerization plays an essential role in the fibrillation processes of the cell wall to produce CNFs and that there is a linear relationship between the degree of polymerization and the length of CNFs, which is independent of the type of enzyme, enzyme charge, and intensity of the applied mechanical treatment. In addition, it is determined that the percentage of the decrease in the degree of polymerization of CNFs due to mechanical treatment is irrespective of the applied enzyme charge. Finally, it is shown that the aspect ratio is a good indicator of the efficiency of the fibrillation process, and is directly related to the mechanical properties of CNF films.

## 1. Introduction

Due to their abundance and sustainability, plant cellulose and cellulosic nanomaterials attract increasing interest as an alternative to synthetic materials, especially as fillers and reinforcements for composite materials [1]. Cellulose nanomaterials (CNM) comprise a broad spectrum of materials produced by the deconstruction of the cell wall. Due to the surprising and promising characteristics of cellulose nanomaterials (biocompatible and transparent properties, excellent mechanical behavior at low weight, and very reactive due to the hydroxyl groups present on their surface [2,3,4]) several researchers focus their interest on the study of this nanomaterial and its potential uses at an industrial level: coating, polymeric reinforcement, 3D printing, rheological modifications, among others [5,6]. The term “cellulose nanofibrils (CNF)” refers to fibrils with nanoscale widths (less than 100 nm) [7], whose average length values are estimated in the order of several micrometers [8].

In previous decades, the challenge associated with the isolation of CNFs was to reduce the high-energy demand required for the mechanical fibrillation process, reported as specific energy values between 5 and 70 kWh/kg [9]. However, with the incorporation of pretreatment methods, such as chemical pretreatments (carboxymethylation, carboxylation, quaternization, sulfonation, and oxidation) or enzymatic pretreatments, energy consumption and production costs decreased, making CNFs a more attractive material for commercial applications [10,11]. However, research continues to focus on optimizing existing techniques and developing alternative methods that can benefit the production process or provide CNFs with new properties [4].

The use of cellulase enzymes as a pretreatment to produce CNFs is one of the most studied aspects since it favors the process of the deconstruction of the cell wall, reduces energy consumption, and facilitates the production of nanofibrils with more homogeneous and controlled dimensions [12,13,14,15]. In addition, with the use of the enzymatic hydrolysis, high yields are favored, the environmental impact is reduced, and it is possible to produce materials suitable for biomedical applications [16].

Cellulases are groups of enzymes that catalyze the breakdown of cellulose polymers into smaller polymer chains, or even cellobiose and glucose. Traditionally, these enzymes are divided into three groups: endoglucanase, exo-β-1,4-glucanase or cellobiohydrolase (CBH), and β-glucosidase (cellobiase) [17]. In terms of CNF production with enzymatic pretreatment, endoglucanases are the most interesting, considering that their action is randomly focused on the amorphous regions of cellulose (specifically on the β-1,4 bonds corresponding to C1 of the first glucose unit and C4 of the after unit), breaking the cellulose chain into polymers of shorter length [13,16]. Nechyporchuk et al. [18] compared CNF production using monocomponent endoglucanases and a mixture of endoglucanase, exo-β-1,4-glucanase, and cellobiase, and showed that monocomponent endoglucanase has a better effect on the separation of nanofibrils while inducing less depolymerization of cellulosic chains.

The degree of polymerization (DP) is defined as the number of times that the monomeric unit that forms the polymer chain repeats [13]. This is an important parameter which evaluates the length of cellulose chains and is frequently used to evaluate produced CNFs [1]. Endoglucanases randomly cleave amorphous regions of cellulose chains and drastically decrease the degree of polymerization, but they slowly release soluble sugars from crystalline regions too [19] (p. 247).

The effect of cellulase enzymes on the degree of the polymerization of cellulose and the mechanical fibrillation process is widely reported [1,12,20,21,22,23]. However, the available information does not allow specific relationships to be established between the enzymatic-mechanical treatment applied, the degree of polymerization, and the characteristics of the CNFs. Therefore, determining the morphological parameters of the CNF (length/diameter) is important to control the production of these nano-objects, minimize morphological damage and determine possible uses or applications based on their characteristics. For example, the work of Zeng et al. [24] showed the importance of the length of CNF when used as strength additives in their paper. The authors propose that if the CNFs are not long enough to join two contiguous fibers, they will not be retained during water drainage or will remain in the fiber matrix without making a difference.

This work aims to establish specific relationships between the intensity of enzymatic treatment, the degree of polymerization of the cellulose, the morphology of CNFs (for a fixed amount of energy), and the tensile strength of the films.

## 2. Materials and Methods

### 2.1. Raw Material

The bleached Kraft hardwood pulp (80% Eucalyptus globulus and 20% Eucalyptus nitens) used to produce CNFs was provided by the company CMPC pulp S.A. (Santa Fe Mill, Chile). Pulp was rehydrated, filtered, and pelleted according to ISO 5263. The chemical characterization of cellulosic fibers was performed through acid hydrolysis based on the method described by Mendonça et al. [25] and Andrade et al. [23].

The enzymatic hydrolysis was performed using the commercial enzyme complex of cellulases Quimizime B, provided by CHT group, Santiago, Chile, which had a higher content of endoglucanases and an activity of 7.75 U/mL enzyme. Enzyme activity was determined according to the methodology of Ghose [26].

### 2.2. Production of Cellulose Nanofibrils

#### 2.2.1. Mechanical-Enzymatic Pretreatment

Thirty grams of the oven-dried pulp of Kraft bleached eucalyptus pulp (BHKP) at 10 wt% were refined in a PFI mill at 4000 revolutions with the aim of increasing the accessibility of the enzyme in the substrate. Then, 0.05% of Quimizime B enzyme was added with respect to the dry weight of the pulp. The first enzymatic pretreatment conditions were: 70 min of reaction, temperature of 48 °C, 5% consistency, pH 5 (adjusted with 0.1M HCl), and constant stirring at 800 rpm with a Stirrer Type BS. After the reaction time, the enzyme was denatured at 80 °C for 20 min. Next, the pulp was refined at 46,000 revolutions in a PFI mill at 10% consistency. After the mechanical treatment, the pulp was separated into 5 samples and subjected to enzymatic treatments with the same conditions as the first enzyme pretreatment, only varying the enzyme percentage of each sample: 0%, 0.025%, 0.05%, 0.075%, and 0.1% ml enzyme/g dry weight of the pulp.

#### 2.2.2. High Pressure Homogenization

Suspensions in the water of the treated fibers were prepared according to the sequence of mechanical and enzymatic pretreatments described in paragraph 2.2.1 at a consistency of 0.5%. These were subjected to a homogenization process at a pressure 700 bar using Gea Niro Soavi Panda Plus 2000 homogenizing equipment (Dusseldorf, Germany) provided with an S-type impact head. The treatment consists of passing the fiber suspension through the equipment 15 times in order to produce 5 types of CNFs, which differed only in the enzyme charge of the second enzyme pretreatment.

**Figure 1:**
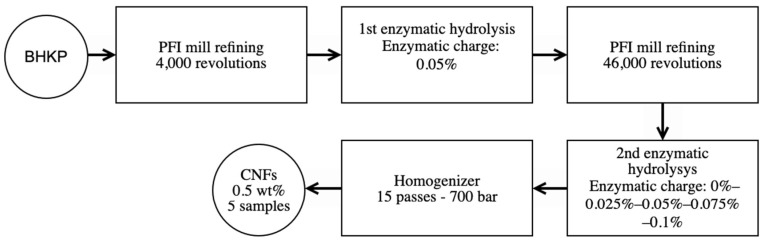
Experimental design flow chart.

Figure 1 shows the flow diagram of the experimental design that summarizes the production of cellulose nanofibrils.

### 2.3. Characterization of Cellulose Nanofibrils

#### 2.3.1. Enzymatic Degradation of Pulp

The enzymatic degradation was evaluated through two indicators: (1) Quantification of carbohydrates present in the filtrate: to determine the effect of the enzyme on cellulosic fibers, the carbohydrates present in the hydrolyzed filtrate were quantified, according to the methodology presented previously [27]. (2) Degree of polymerization: the degree of polymerization of cellulose was determined by measuring the intrinsic viscosity in cuproethylene diamine, according to Chakraborty et al. [28].

#### 2.3.2. Transmittance of CNF Dispersions

The transmittance was determined on CNF dispersions at 0.1%, using a Genesys UV10 spectrophotometer (Thermo Fisher Scientific, Madison, WI, USA) at a wavelength of 800 nm, using distilled water as a reference, according to the methodology presented by Delgado-Aguilar [13].

#### 2.3.3. Intrinsic Viscosity

Intrinsic viscosity was determined from the viscosities of CNF dispersions, according to Albornoz-Palma et al. [22]. For measurements, a Brookfield LVDV-I + viscometer (Middleborough, MA, USA) was used with a ULA (Ultra Low Adapter) spindle, and its configuration corresponded to a double-cylinder geometry, from samples at different concentrations (0.02% (*w*/*v*) to 0.08% (*w*/*v*)). Each sample was heated in a Julabo SW22 thermal bath at 23 °C for 2 h before the measurement. Samples did not show thixotropic behavior and reached a steady state in less than 20 s. The measurement conditions were 23 °C with a shear rate of 73.38 s^−1^.

#### 2.3.4. Morphological Characteristics of CNFs

The average length of CNF dispersions ($\bar{L}$) was measured using S3500 Laser Diffraction Particle Size Analyzer (Microtrac Inc., Montgomeryville, PA, USA) (refractive index: 1.54 [29]) from dispersions at 0.04% (*w*/*v*) consistency, according to Albornoz-Palma et al. [22]. Each sample was sonicated for 60 s in the equipment before measuring to eliminate the formation of entanglements and aggregates.

The aspect ratio ($\bar{L}/\bar{d}$) and the average width ($\bar{d}$) were calculated from the relationship between the intrinsic viscosity CNF dispersions ([η]) and the average length of CNFs [22]:

(1) $$\rho \left[\eta \right]=0.051\;p^{1.85}$$

where ρ is the density of CNFs (1.6 g/mL) and p  is the aspect ratio of CNFs ($\bar{L}/\bar{d}$).

#### 2.3.5. Mechanical Properties of CNF Films

The maximum load stress (N) supported by rectangular samples (2 cm wide, 8 cm long, and 0.025 cm thickness) cut from CNF films 100 g/m^2^ made by solvent casting was determined by the tension testing equipment (TestResources Inc, Shakopee, MN, USA). The tests were performed on 8 specimens for each sample, with a 30 mm/min crosshead speed, and the clamp span was set to 40 mm.

## 3. Results and Discussions

### 3.1. Mechanical-Enzymatic Pretreatment

Cellulolytic enzymes are glucoside hydrolases that break the β-(1→4)-glucosidic bonds of carbohydrates by the inversion or retention of the anomeric carbon configuration [19].

Table 1 shows the percentage distribution of the solid residue and the fraction of solubilized carbohydrates from the eucalyptus pulp after the enzymatic pretreatments. As expected, the enzyme complex solubilized part of the hemicelluloses and cellulose of the raw material, whose initial chemical composition was: 77.7 ± 0.5% of cellulose, 21.1 ± 0.5% of hemicellulose and, <1% of lignin, which coincided with what was reported by other authors for this type of pulp [15,23,30]. The first enzymatic treatment (P_E_), which was identical for the 5 CNFs, solubilized a lower amount of cellulose and xylan than the pulps with double enzymatic treatment, hence the carbohydrate yield in the solid fraction was higher. This was due to the fact that in the first enzyme treatment, the accessibility of the enzyme to the substrate was lower than the second. Both the mechanical refining process, as well as the first enzymatic treatment, caused a greater internal and external fibrillation of the fibers, increasing the exposed surface area of the fiber and the absorption of water, which facilitated the accessibility of the enzyme for the second hydrolysis enzymatic.

The percentage of carbohydrates in the solid fraction decreased as the enzyme charge of the second enzyme treatment increased from 0% to 0.1%, which was mainly due to the hydrolysis of amorphous cellulose [12]. Furthermore, in the second enzymatic treatment, the degradation of xylans was statistically the same for all samples, so the difference in the decrease in solid yields was clearly due to the hydrolysis of cellulose.

### 3.2. Morfological Characteristics of the CNFs

Determining the morphological characteristics of CNFs is relevant to understanding the final properties of this nanomaterial. Table 2 shows the morphological characteristics of the CNFs produced from pulps with different enzyme charges and the same mechanical treatment. Regarding the width, length, and degree of polymerization of the CNFs, a decrease in these characteristics is observed as the enzyme charge increases, which can be attributed to a greater hydrolysis of the cellulose chains due to an increased enzyme concentration. Endoglucanases drastically decrease the degree of polymerization, which can increase the frequency of the rupture or breakpoints within the fibers, facilitating the access of water to the interior of the cell wall, and consequently, the process of mechanical fibrillation.

The transmittance of light at a specific wavelength through a CNF suspension indicates the presence of smaller and/or more homogeneous nano-objects and is often used as an indirect method to estimate the degree of fibrillation of CNF dispersions [13,14]. According to the above, the results of Table 2 show that the increase in the enzyme charge effectively allows for the greater fibrillation of the BHKP, which is reflected in the decrease in the lengths and widths of the CNFs. From a technological point of view, it is often interesting to produce fibrils with morphological characteristics that favor certain applications. For example, a higher aspect ratio is expected to favor the reinforcing properties of composite materials.

When observing the aspect ratio of the nanofibrils (Table 2), it can clearly be seen that this parameter does not have the same tendency as the transmittance of light and that its variation is affected by the enzyme charge during treatment, increasing with the enzyme charge to a maximum value and then decreasing.

**Figure 2:**
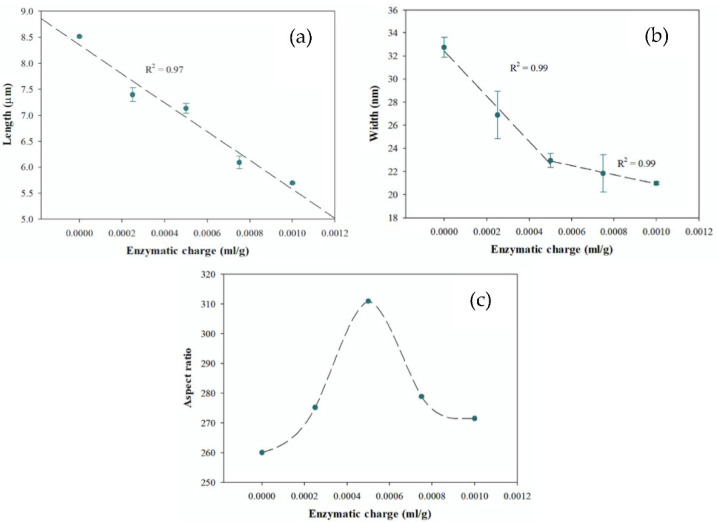
Morphological characteristics of the CNFs as a function of the enzyme charge in the second enzyme pretreatment: (**a**) length, (**b**) width, and (**c**) aspect ratio.

With the aim of gaining a better understanding of how the enzyme charge affects the morphological characteristics of CNFs, the relationships are shown in Figure 2.

For the length of the CNFs (Figure 2a), a negative linear relationship with the enzyme charge is observed, whose differences are statistically significant between the different samples (LSD method, 95% confidence). These results prove that a higher enzyme charge can generate significant changes in the lengths of the CNFs for the same mechanical treatment. In this case, a charge of 0.1% for an enzyme (in the second enzymatic treatment) generates a 33% decrease in CNFs lengths.

As mentioned by Taheri and Samyn [31], the minimum width of the fibrils produced during fibrillation depends on the operating conditions and the equipment used for mechanical fibrillation. When analyzing the widths of the CNFs (Figure 2b), it is observed that there is a drastic and significant decrease (*p*-value < 0.05) at low enzyme charges (<0.0005 mL/g). However, for enzyme charges greater than 0.0005 mL/g, the decrease in width is much less pronounced, and there are no statistically significant differences (LSD method, 95% confidence). These results suggest that, for the same mechanical treatment, an increase in the fibrillation of the enzyme charge facilitates up to the minimum width given by the equipment. In this sense, there is evidence for the need to define a control parameter that encompasses the morphological properties of CNFs and optimizes the enzymatic pretreatments for each mechanical process.

The morphological parameter that best reflects the concept of fibrillation corresponds to the aspect ratio of the CNFs, since it is shown that an increase in the enzyme charge favors fibrillation as a result of the transversal break of the fibrils, which reduces the length of the fibrils, and the longitudinal break decreases their width until a minimum value is obtained. Consequently, the aspect ratio of the CNFs presents a maximum value (*p* = 311) (Figure 2c) for an enzyme charge in the second enzyme treatment, of 0.0005 mL/g.

An efficient fibrillation process seeks to produce a homogeneous material with small widths (<50 nm) and attempts to decrease the transversal break of the fibrils (length). Therefore, the aspect ratio is the parameter that best reflects these characteristics and corresponds to a good indicator for the effectiveness of the fibrillation process.

On the one hand, Andrade et al. [23] determined that, for CNFs produced from the same raw material and with the same enzymatic-mechanical treatment, the aspect ratio was 330. On the other hand, in their study, Albornoz-Palma et al. [22] produced CNFs with an aspect ratio of 303, from the same raw material and mechanical treatment, but with a different type and charge of enzyme.

### 3.3. Effect of Mechanical-Enzymatic Treatment on the Degree of Polymerization of Cellulose

The degree of polymerization is an important parameter that evaluates the length of cellulose chains and is frequently used to evaluate CNFs [1]. Table 3 shows the variation in the degree of polymerization as a function of the applied treatment. As mentioned above, endoglucanase enzymes are characterized by a rapid decrease in the degree of polymerization [19].

The “Variation 1” in Table 3 indicates the decrease in DP regarding the initial raw material (P), which decreases as the intensity of the mechanical and/or enzymatic pretreatment applied increases. The DP of cellulose decreases between 72 and 81% for the CNFs produced, with DP values between 278 and 447, which coincides with those reported by various authors who produced CNFs with similar enzymatic and mechanical pretreatments. For example, in their study, Albornoz-Palma et al. [22] used cellulase enzymes (1.2 wt%) to produce CNFs with a degree of polymerization of 228; Tarres et al. [14] used endoglucanase enzyme (0.032%) to produce CNFs with a DP of 309, and Andrade et al. [23] used the same raw material and enzyme cocktail, with a charge of 0.1% of the enzyme in two treatments (0.05% each) producing CNFs with a DP of 303, causing a decrease of 75% with respect to the initial raw material. Additionally, Qing et al. [1] used a different mechanical treatment (SuperMassCollider-Microfluidization 15 passes at 1500 bar) and produced CNFs with a DP of 280 when dosing 3 FPU of enzyme/g fiber. From the above, it seems that the lower limit of the degree of polymerization for CNFs is 220, with enzymatic pretreatment having a high degree of fibrillation.

The “Variation 2” in Table 3 represents the decrease in the DP of the CNFs after the homogenization process with respect to the pulps with mechanical-enzymatic pretreatments. The results show that the decrease in DP due to the homogenization process is independent of the enzyme charge since, for the same mechanical treatment (15 passes through the homogenizer) the decrease in DP is close to 40% for all cases.

**Figure 3:**
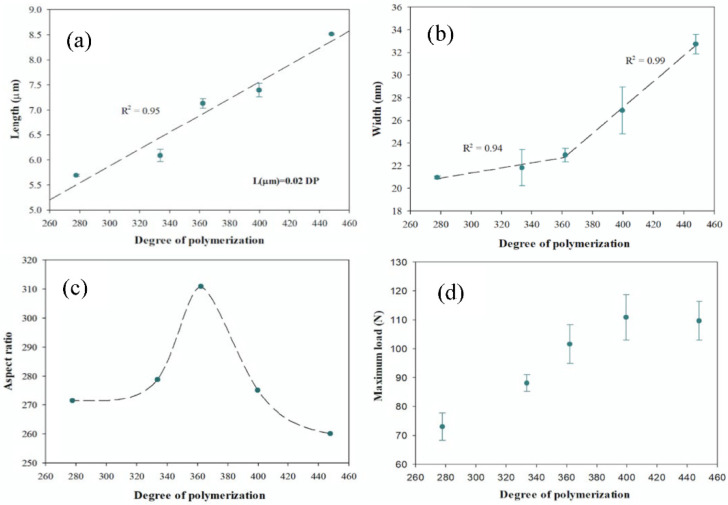
Morphological characteristics of CNFs and resistance of CNF films depending on the degree of polymerizationL: (**a**) length, (**b**) width, (**c**) aspect ratio, and (**d**) maximum load.

Figure 3 shows the change in the morphological characteristics of the CNFs and the resistance of the CNF films as a function of the DP of cellulose.

The length of the CNFs (Figure 3a) is linearly related to the degree of polymerization, with a determination coefficient of 0.95. This line relationship of L (µm) = 0.02 DP coincides with the results of the study by Albornoz-Palma et al. [22], which produced CNFs with enzymatic pretreatment but used another enzymes cocktail of cellulase enzymes and a higher enzyme charge (0.012 mL/g). Furthermore, these authors related the degree of polymerization of their samples to a different number of passes through the homogenizer (0, 1, 2, 4, 7, 10, 15), showing the same linear regression. Thus, in view of the above, the length of the CNFs and the DP are linearly related, independent of the type of cellulase enzyme, enzymatic charge, and intensity of the mechanical treatment applied in the homogenizer.

As the relationship between the length and degree of polymerization is positively linear (Figure 3a) and the relationship between the length and enzyme charge is negatively linear (Figure 2a), width and aspect ratio show the same trends as a function of the degree of polymerization, as well as with the enzymatic charge, but in a more specular way. Because of the above, the width of the CNFs (Figure 3b) at a DP of less than 362 does not show statistically significant differences (LSD method, 95% confidence). On the other hand, at a DP of greater than 362, the differences are significant, and the DP of the samples varies by up to 38%. The relationship between the aspect ratio and the DP has a maximum at DP = 362 (Figure 3c). This maximum value is due to the fact that with a DP of less than 362, there is a change in the lengths of the CNFs but no change in the widths, so the aspect ratio decreases.

In the literature, it is observed that a higher degree of polymerization is strongly related to the improvement of the mechanical properties of nanofibrils [13,32]. Regarding the maximum load supported by the CNF films (Figure 3d), it is observed that, for DP values greater than 362, there are no statistically significant differences (Bonferroni method, 95% confidence). However, the maximum load decreases for DP by less than 362, and the values are statistically different (Bonferroni method, 95% confidence). When relating these results to the enzymatic charge and the aspect ratio, the inflection point, where the maximum load supported by the CNF films begins to decrease, corresponds to the point where the aspect ratio is at a maximum value. This maximum aspect ratio is provided by the equipment used (homogenizer in this case), where for the high enzymatic charge (for the same mechanical process), the transverse rupture of the fibers is favored; thus, the aspect ratio decreases.

## 4. Conclusions

The enzymatic pretreatment facilitates the deconstruction of the cell walls of the fibers. However, at high enzyme charges, the aspect ratio of the CNFs shows a drastic decrease as a result of inefficient fibrillation due to the transversal break of CNFs.

Moreover, it is shown that the DP is linearly related to the length of the CNFs, independent of the type of cellulase enzyme, enzymatic charge, and intensity of the mechanical treatment applied in the homogenizer. Furthermore, for the same intensity in the mechanical treatment, the percentage decrease in DP is independent of the enzymatic charge.

Finally, the maximum load supported by CNF films is linearly related to the DP up to a maximum value, corresponding to the maximum value of the aspect ratio. Therefore, the aspect ratio is a good indicator of the efficiency of the fibrillation process, and it is directly related to the mechanical properties of CNF films.

## Figures and Tables

**Table 1:** Enzymatic hydrolysis and solubilization of carbohydrates.

Pulp	Sample	Solubilized Compounds		Solid Carbohydrate Yield (%)
		Cellulose (% Initial Cellulose)	Xylan (% Initial Xylan)	
*Eucalyptus*	P_0_	1.9 ± 0.1	1.5% ± 0.3	96.6 ± 0.4
	P_0.025_	3.1 ± 0.1	3.4% ± 0.2	93.5 ± 0.2
	P_0.050_	3.6 ± 0.2	3.2% ± 0.2	93.2 ± 0.3
	P_0.075_	4.3 ± 0.1	3.2% ± 0.2	92.5 ± 0.1
	P_0.1_	4.6 ± 0.1	3.1% ± 0.1	92.3 ± 0.2

**Table 2:** Morphological characteristics of CNFs.

	Length (μm)	Width (nm)	Aspect Ratio	Transmittance (%)	Degree of Polymerization
CNF_0_	8.5 ± 0.1	32.7 ± 0.9	260.0	73.6	447
CNF_0.025_	7.4 ± 0.3	26.9 ± 2.1	275.0	74.1	400
CNF_0.050_	7.1 ± 0.2	22.9 ± 0.6	310.9	77.1	362
CNF_0.075_	6.1 ± 0.2	21.8 ± 1.6	278.9	78.5	334
CNF_0.1_	5.7 ± 0.1	21.1 ± 1.1	271.5	85.9	278

**Table 3:** Variation in the degree of polymerization depending on the treatment applied.

Sample	Degree of Polymerization	Variation 1	Variation 2
P	1438	---	
PR	1231	−14%	
PE	916	−36%	
P_0_	760	−47%	
P_0.025_	649	−55%	
P_0.05_	601	−58%	
P_0.075_	529	−63%	
P_0.1_	497	−65%	
CNF_0_	447	−69%	−41%
CNF_0.025_	400	−72%	−38%
CNF_0.050_	362	−75%	−40%
CNF_0.075_	334	−77%	−37%
CNF_0.100_	278	−81%	−44%

## Data Availability

Data sharing is not applicable to this article.

## Nomenclature

P	BHKP
P_R_	BHKP—4000 rev PFI
P_E_	BHKP—4000 rev PFI—0.05%
P_0_	BHKP—4000 rev PFI—0.05%–46,000 rev PFI—0%v
P_0.025_	BHKP—4000 rev PFI—0.05%–46,000 rev PFI—0.025%
P_0.050_	BHKP—4000 rev PFI—0.05%–46,000 rev PFI—0.05%
P_0.075_	BHKP—4000 rev PFI—0.05%–46,000 rev PFI—0.075%
P_0.1_	BHKP—4000 rev PFI—0.05%–46,000 rev PFI—0.1%
CNF_0_	BHKP—4000 rev PFI—0.05%–46,000 rev PFI—0%—15 pass
CNF_0.025_	BHKP—4000 rev PFI—0.05%–46,000 rev PFI—0.025%—15 pass
CNF_0.050_	BHKP—4000 rev PFI—0.05%–46,000 rev PFI—0.050%—15 pass
CNF_0.075_	BHKP—4000 rev PFI—0.05%–46,000 rev PFI—0.075%—15 pass
CNF_0.100_	BHKP—4000 rev PFI—0.05%–46,000 rev PFI—0.1%—15 pass
